# Abcès sous périoste orbitaire compliquant une sinusite: à propos d'un cas

**DOI:** 10.11604/pamj.2014.18.128.4412

**Published:** 2014-06-10

**Authors:** Hakima Elouarradi, Rajae Daoudi

**Affiliations:** 1 Université Mohammed V Souissi, Service d'Ophtalmologie A de l'Hôpital des Spécialités, Centre Hospitalier Universitaire, Rabat, Maroc

**Keywords:** Abcès, périoste, sinusite, Abcès, périoste, sinusite, abscess, periosteum, sinusitis

## Image en medicine

Les complications orbitaires d'origine sinusienne sont rares mais graves, pouvant mettre en jeu le pronostic fonctionnel et vital. Elles constituent une urgence diagnostique et thérapeutique. Le traitement comporte toujours un volet médical basé sur une antibiothérapie adaptée, parfois associé à un traitement chirurgical. Nous rapportons l'observation d'un enfant âgé de 8 ans, emmené par ses parents aux urgences suite à l'apparition 3 jours avant d'une exophtalmie inflammatoire de l’œil droit d'installation brutale (A). On note la notion de rhinorrhée purulente associée à une fièvre. L'examen ophtalmologique retrouve une bonne acuité visuelle de loin corrigée à 10/10 au niveau des 2 yeux, avec au niveau de l’œil droit, un chémosis conjonctival, et une ophtalmoplégie totale (B). L'examen des segments antérieur et postérieur est sans particularités. Une tomodensitométrie orbitocérébrale réalisée en urgence a confirmé l'existence d'un abcès collecté sous périoste intra orbitaire droit associé à un pan sinusite (C,D). Devant cet aspect typique, un drainage sous anesthésie générale est réalisé par canthotomie médiale (E) et une antibiothérapie intraveineuse a été instaurée en urgence, associée à une corticothérapie per os de courte durée instaurée à j3 de l'antibiothérapie. L’évolution a été marquée par la bonne amélioration clinique avec régression de l'exophtalmie et des signes inflammatoires (F, G). L'infection orbitaire est une urgence. Il est important qu'elle soit reconnue précocement et traitée de façon énergique. C'est toujours une cause possible de cécité, voire de mortalité en cas de complication(s).

**Figure 1:**
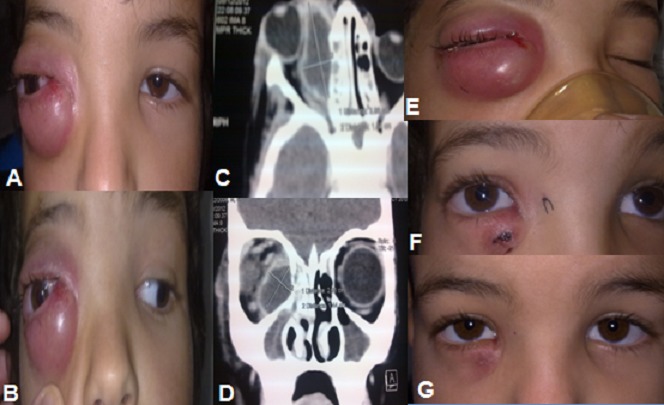
(A) et (B) Exophtalmie inflammatoire d'installation brutale droite; (C) et (D) Aspect TDM orbitaire objectivant l'aspect typique d'abcès sous périoste orbitaire droit; (E) Aspect per opératoire; (F) et (G) Bonne amélioration clinique après drainage et traitement médical

